# Grammatical analysis as a distributed neurobiological function

**DOI:** 10.1002/hbm.22696

**Published:** 2014-11-24

**Authors:** Mirjana Bozic, Elisabeth Fonteneau, Li Su, William D Marslen‐Wilson

**Affiliations:** ^1^ Department of Psychology University of Cambridge Downing Street Cambridge, United Kingdom; ^2^ MRC Cognition and Brain Sciences Unit 15 Chaucer Road Cambridge United Kingdom

**Keywords:** brain, grammar, computation, hemispheric distribution

## Abstract

Language processing engages large‐scale functional networks in both hemispheres. Although it is widely accepted that left perisylvian regions have a key role in supporting complex grammatical computations, patient data suggest that some aspects of grammatical processing could be supported bilaterally. We investigated the distribution and the nature of grammatical computations across language processing networks by comparing two types of combinatorial grammatical sequences—inflectionally complex words and minimal phrases—and contrasting them with grammatically simple words. Novel multivariate analyses revealed that they engage a coalition of separable subsystems: inflected forms triggered left‐lateralized activation, dissociable into dorsal processes supporting morphophonological parsing and ventral, lexically driven morphosyntactic processes. In contrast, simple phrases activated a consistently bilateral pattern of temporal regions, overlapping with inflectional activations in L middle temporal gyrus. These data confirm the role of the left‐lateralized frontotemporal network in supporting complex grammatical computations. Critically, they also point to the capacity of bilateral temporal regions to support simple, linear grammatical computations. This is consistent with a dual neurobiological framework where phylogenetically older bihemispheric systems form part of the network that supports language function in the modern human, and where significant capacities for language comprehension remain intact even following severe left hemisphere damage. *Hum Brain Mapp 36:1190–1201, 2015.* **© 2014 The Authors Human Brain Mapping Published by Wiley Periodicals, Inc.**

## INTRODUCTION

Converging neuroimaging and neuropsychological evidence suggest that language comprehension reflects a coalition of processing functions distributed over two neurobiologically distinct systems. The first is a distributed bihemispheric system, involved in the lexical, semantic, and pragmatic interpretation of auditory inputs. The second is a left‐lateralized frontotemporal system, activated by core grammatical computations in the domain of inflectional morphology and syntax [Bozic et al., 2010; Marslen‐Wilson and Tyler, 2007]. Recent data from brain damaged patients suggest, however, that the bihemispheric system may also have a significant role in supporting aspects of sentence processing [Tyler et al., 2010; Wright et al., 2012]. This raises the question of how—and whether—specifically grammatical capacities are distributed over these two systems. We investigated this by comparing the processing signatures of inflectionally complex words and minimal phrases, two instances of grammatical combination that may nonetheless vary in their neurocognitive substrates.

### Neurobiology of Language Processing

In the context of human language comprehension, the bihemispheric system supports the ability to identify the words a speaker is producing—typically by integrating auditory and visual cues in face‐to‐face interaction—but also to make sense of these word meanings in the general context of the listener's knowledge of the world and the specific context of speaking. Evidence comes from both neuroimaging and neuropsychology. Neuroimaging studies with healthy participants show the involvement of bilateral superior, middle, and inferior temporal areas in language comprehension [Hickok and Poeppel, 2007], often with little evidence of left frontal activation [e.g., Tyler et al., 2010]. Lesion studies show that dynamic access to lexical meaning, and the ability to construct semantic and pragmatic interpretations of incoming speech, can remain largely intact even after extensive damage to left frontal and posterior temporal regions [Caramazza and Zurif, 1976; Caplan et al., 1996; Miceli et al., 1983; Marslen‐Wilson and Tyler, 1997; Tyler et al., 2011; Wright et al., 2012].

The aspects of language function that critically depend on intact left frontotemporal circuits are those related to grammatical processing, specifically in the domain of inflectional morphology and syntax. Patients with left hemisphere damage (especially the left inferior frontal gyrus [LIFG]) have problems with the processing of regularly inflected words (e.g., *played*), formed by combining stems and grammatical suffixes [Longworth et al., 2005]. The involvement of left frontotemporal regions in processing inflectionally complex words is also supported by substantial neuroimaging evidence in healthy participants, across languages and imaging modalities [Bozic et al., 2010; Lehtonen et al., 2006; Shtyrov et al., 2005; Szlachta et al., 2012]. This left‐lateralized activation arguably reflects the computational demands associated with the combinatorial implications of the inflectional morpheme, once identified. Consistent with this, inflected verbs trigger more left frontotemporal activation than inflected nouns, reflecting the fact that verbs have rich morphosyntactic paradigms that engage combinatorial structure‐building processes more strongly than nouns when these processes are triggered by the presence of an inflectional morpheme [Longe et al., 2007; Tyler et al., 2008].

Within the broader syntactic domain, there is unambiguous evidence for a strong link between LH perisylvian regions and complex syntax. Numerous neuropsychological studies show that left hemisphere patients have particular problems with aspects of sentential processing involving complex syntactic structures such as passive constructions, embedded clauses, or long‐distance dependencies [Caramazza and Zurif, 1976; Caplan et al., 1996]. Neuroimaging studies with unimpaired adult populations corroborate this evidence, showing that LIFG increases its activity as a function of the structural complexity of well‐formed sentences [Friederici et al., 2006b; Makuuchi et al., 2009] and is implicated in the incremental unification of structured sequences [Hagoort, 2013]. The patterns of activation for processing complex syntax show substantial overlap with those seen for regular inflections, typically involving Brodmann areas 44/45 linked to L posterior temporal regions, suggesting the engagement of overlapping computational mechanisms.

Notably, however, damage to left frontotemporal regions does not necessarily affect patients' ability to understand sentences in which familiar words are presented in canonical (English) subject‐verb‐object word order. For instance, left‐hemisphere patients asked to match spoken sentences to sets of pictures will correctly match the semantically reversible sentence “The woman pushed the girl” to a picture of a woman pushing a girl. At the same time, however, they will incorrectly match the passive sentence “The woman is being pushed by the girl” to the same picture [Schwartz et al., 1980; Tyler et al., 2011]. This pattern is characteristic of so‐called “asyntactic comprehension,” where patients can use the meanings of words and local cues to grammatical relations to understand spoken utterances [Ostrin and Tyler, 1995], but where this process breaks down when more complex reordering of syntactic relations is required. This accumulating evidence that the bihemispheric system can support some degree of sentence comprehension even in the presence of LH damage raises basic questions about the nature of these capacities, how far they are specifically grammatical, and how they might contrast with the more complex combinatorial grammatical computations that the left hemisphere is claimed to support.

To address this question, we need to distinguish between potentially different types of combinatorial mechanisms argued to contribute to grammatical computations. The existing literature offers several—often contradictory—perspectives. One prominent view [Chomsky, 1956; Fitch and Hauser, 2004; Fitch and Friederici, 2012; Levelt, 1974] holds that grammatical processes involve coordinated mechanisms of linear computations, which specify simple left‐to‐right transitional probabilities between adjacent elements (ABAB), and nonlinear computations, which allow for construction of complex hierarchies by, for example, embedding strings into other strings (A[AB]B). These two types of grammatical computation have been associated with two distinct left hemisphere architectures: L frontal operculum (FOP) and anterior STG for linear computations, and L BA 44/45 and posterior STG for the nonlinear ones [Friederici et al., 2006a; Friederici, 2011]. A contrasting view put forward by Hagoort, Petersson, and coworkers [Hagoort, 2013; Petersson and Hagoort, 2012; Petersson et al., 2012] rejects this distinction, arguing instead for a single mechanism of incremental sequence processing, modified by the differences in memory requirements for simple and complex strings. This process is primarily related to the left inferior frontal regions (BA 44/45). Another recent view [Bornkessel‐Schlesewsky and Schlesewsky, 2013] distinguishes between the time‐independent unification of conceptual representations and time‐dependent syntactic structure building, linked to LH ventral and dorsal pathways, respectively.

One feature that is common to all current proposals is a strong emphasis on the left‐lateralization of the relevant neurocognitive processing mechanisms. The evidence for this strict left‐lateralization is, however, somewhat mixed. While the link between computations involved in processing complex syntax—however conceptualized—and left frontotemporal regions seems unambiguous, the processing (and violation) of local grammatical structures activates regions in right temporal lobes in addition to the FOP and L STG/MTG [Friederici et al., 2000a; Friederici et al., 2003; Friederici et al., 2006a; Ni et al., 2000]. Similarly, MEG evidence reveals bilateral temporal engagement for early syntactic parsing, argued to support the construction of simple syntactic structures based on word category information [Friederici et al., 2000b]. This raises the possibility of bihemispheric involvement in the computation of local grammatical structure, and is consistent with evidence more generally that the processing of syntactically simple and canonical utterances need only involve bilateral temporal structures [Friederici et al., 2000a; Tyler et al., 2010].

Taken together, the existing findings raise more questions than answers regarding the distribution of combinatorial grammatical processes within the language system, and the underlying computations they reflect. We address these questions in this study by comparing the processing signatures for inflectionally complex words and minimal phrases, contrasting these with grammatically simple forms such as *dog* or *house*. Linguistically, both inflectional and phrasal complexity implicate combinatory grammatical operations, where the relevant linguistic elements need to be combined and interpreted. As discussed, regular inflections in English are combinations of stems like *jump* with suffixes like *–ed* or *‐s* that modify the grammatical properties of the stem, and that engage with the structural interpretation of the string in which they are potentially embedded. Short phrases (e.g., *the rug, I follow*) are minimally complex sequences of words combined according to grammatical structure‐building constraints. On currently dominant views of core linguistic capacities, the processing of both inflected forms and short phrases should rely on combinatorial mechanisms supported by the left hemisphere systems [Berwick et al., 2013; Friederici, 2011]. More specifically, these accounts would predict that inflections will activate dorsal LIFG (BA 44/45) and posterior temporal regions; while simple phrases—involving only local dependencies—should activate more ventral IFG/the FOP, as well as left anterior temporal lobe. The neuropsychological and neuroimaging data reviewed above, however, suggest that some of these mechanisms might be supported by a more distributed bilateral processing network.

To focus and strengthen the investigations of the neurocognitive distribution of grammatical computations, we added a manipulation of the grammatical category of the stems to which inflectional and phrasal structure were applied. As noted above, verbs have richer morphosyntactic paradigms and engage combinatorial processes more strongly than nouns—but only when placed in the appropriate grammatical environments. Longe et al. [2007], for instance, compared the processing of nouns and verbs presented as bare stems (e.g., *snail, hear*) and as inflected forms (e.g., *snails, hears*), and found that verb inflections activated LH regions more strongly than noun inflections while there were no differences between verb and noun stems. Consistent with this, Thompson et al. [2007] showed increased LH activity for verbs with richer argument structure, which are arguably more combinatorially complex. These considerations, coupled with the pervasive category ambiguity of word stems in English (most stems can either function as verbs or nouns), made it both necessary and informative to control the grammatical category of the stems used in the experiment.

Accordingly, four categories of stems were chosen, based on their frequency of occurrence as verbs or nouns: unique verbs (e.g., *sing*), verb dominant stems (e.g., *wash*), noun dominant stems (e.g., *cough*), and unique nouns (e.g., carrot). These were combined with the inflectional suffix ‐s to create plural nouns or third‐person singular verbs; and with either a personal pronoun (*I, you, we*) or an article (*a, an, the*) to create verb or noun phrases. If the engagement of the LH system for inflected forms primarily reflects their combinatorial implications, then left‐lateralized effects should be stronger for the two verb conditions than for the two noun conditions. If phrasal combination engages LH mechanisms in the same way, then similar verb/noun contrasts should be seen here as well. The stem conditions should not show these effects, as previously reported by Longe et al. [2007] and Tyler et al. [2008].

In summary, the current experiment investigated the neural signatures of three different types of linguistic materials—bare stems, inflected forms and phrases—paired with the manipulation of the grammatical properties of the constituent stem along the verb‐noun dimension. This created 12 test conditions, which were presented alongside a musical rain (MuR) baseline that shares the complex auditory properties of speech but does not trigger a speech percept [Uppenkamp et al., 2006]. To minimize confounds due to task‐related activation, participants listened to the stimuli passively and performed an occasional one‐back memory task.

To compare the neural distribution of the grammatical computations triggered by these conditions, we analyzed the data using both standard univariate measures and the multivariate Representational Similarity Analysis (RSA) technique [Kriegeskorte et al., 2008]. In contrast to univariate methods, which are driven by variation in overall levels of regional activation, RSA is sensitive to the patterning of neural activity across multiple voxels and provides more qualitatively specific data about the type of information processed in a given brain area.

## MATERIALS AND METHODS

### Participants

Participants were 18 right‐handed native speakers of British English, screened for neurological or developmental disorders. All gave informed consent and were paid for their participation. The study was approved by the Peterborough and Fenland Ethical Committee.

### Stimuli

A set of 160 verbs and nouns were presented three times each; once as a bare stem (e.g., *sing, rug*), once as an inflected form (e.g., *sings, rugs*), and once as a short phrase (e.g., *I sing, a rug*), for a total of 480 test items. All inflected forms were constructed by adding the suffix –s to the end of the stem. Phrases were constructed by adding a personal pronoun (*I, you, we*) to verb stems, or an article (*the, a, an*) to noun stems. Stems were matched on a range of psycholinguistic variables: length, number of phonemes, lemma and word form frequency and familiarity (all *P* > 0.1). They were further controlled for verb dominance, based on their frequency of occurrence as a verb or a noun. The dominance measure was calculated as a ratio of verb/noun frequency for each stem obtained from the CELEX database [Baayen et al., 1995], and expressed on a scale 0–1. Based on the dominance measure, stems were divided into four categories: verb unique (stems that are always used as verbs, e.g., *sing*; mean dominance = 1; *N* = 40); verb dominant (stems that are more commonly used as verbs than nouns, e.g. *wash*; mean dominance = 0.84; *N* = 40); noun dominant (stems that are more commonly used as nouns than verbs, e.g., *cough*; mean dominance = 0.06; *N* = 40); noun unique (stems that are always used as nouns, e.g., *carrot*; mean dominance = 0; *N* = 40). Dominance values were significantly different across the four conditions, as well as between verb unique and verb dominant sets, and noun unique and noun dominant sets (all *P* < 0.001). Stems that can be used as verbs (categories 1–3) were matched on their combinatorial complexity (number of complements they can take and the entropy of this distribution, both *P* > 0.1), using the Valex lexicon (http://www.cl.cam.ac.uk/~alk23/subcat/lexicon.html).

The 480 test words were mixed with 240 acoustic baseline trials and 240 silence trials. The acoustic baseline trials were constructed to share complex auditory properties of speech without triggering phonetic interpretation. They are produced by extracting the temporal envelope for 80 stems, 80 inflected forms, and 80 phrases, and then filling them with musical rain (MuR), constructed by jittering the frequency and periodicity of 10 ms fragments of vowel formants [Bozic et al., 2010; Uppenkamp et al., 2006]. The resulting envelope‐shaped MuR stimuli are length‐matched to the speech stimuli and have root mean squared level and long‐term spectrotemporal distribution of energy matched to the corresponding speech stimuli but do not trigger a speech percept.

### Procedure

To avoid task‐related confounds, we used a passive listening paradigm with an occasional one‐back memory task. Participants were instructed to listen carefully to each sound and on 5% of trials respond whether the sound they were currently hearing was the same as the previous one. They indicated their responses by button press with their right hand (same = YES, different = NO). Only task‐free trials were subsequently analyzed.

There were four blocks of 280 items each, pseudorandomized with respect to their type (stem, inflected, phrase, baseline, null, task). Five dummy items at the beginning of each block allowed the signal to reach equilibrium. The experiment started with a short practice session outside the scanner, where participants were given feedback on their performance.

Scanning was performed on a 3T Trio Siemens Scanner at the MRC‐CBU, Cambridge, using a fast sparse imaging protocol to minimize the interference of scanner noise with auditory processing (gradient‐echo EPI sequence, TR = 3.4 s, TA = 2 s, TE = 30 ms, flip angle 78 degrees, matrix size 64 × 64, FOV = 192 × 192 mm, 32 oblique slices 3‐mm thick, 0.75‐mm gap). MPRAGE T1‐weighted scans were acquired for anatomical localization. With the TR of 3.4 s (consisting of 2 s volume acquisition and 1.4 s silence) and 280 items per block, each block was just under 16 min in length. Block order was counterbalanced across participants. Stimuli were presented within the 1.4 s silence period between scans, and at least 100 ms after the offset of the previous scan to avoid perceptual overlap between the stimulus and the scanner noise. The time between the offset of one stimulus and the beginning of another varied between 2.5 and 3 s.

Data were analyzed using conventional univariate techniques, as well as multivariate Representational Similarity Analyses implemented in the RSA toolbox [Nili et al., 2014]. The analyses focused on the distributed bilateral language processing network as identified by the literature and in our own work [Bozic et al., 2010; Friederici, 2011; Hickok and Poeppel, 2007; Tyler et al., 2005]. This network covered bilateral temporal lobes (superior, middle and inferior temporal gyri, temporal poles), inferior frontal gyri (BA 44, BA 45, BA 47), and FOP. Regions were defined anatomically using WFU Pickatlas, or based on published coordinates for FOP, where an anatomical definition was not available. FOP was defined as a 10 mm sphere centered on MNI coordinates −36 20 −3, following Friederici et al. [2006a] and Lohmann et al. [2010]. The right hemisphere FOP was defined in the same way, with the coordinates 36 20 −3.

For both analyses, preprocessing was performed in SPM5 using the Automatic Analysis library (https://github.com/rhodricusack/automaticanalysis). For the univariate analyses, this involved image realignment to the first EPI image to correct for movement, segmentation, and spatial normalization of functional images to the MNI reference brain, and smoothing with an 10 mm isotropic Gaussian kernel. Slice‐timing correction was not applied as the interpolation is unlikely to be accurate due to the discontinuous nature of sparse‐sampling. The data for each subject were analyzed using the general linear model, with motion regressors as covariates of no interest to account for any residual movement artefacts. A high‐pass filter with a 128 s cut‐off was applied to remove low‐frequency noise. The first‐order autoregressive model (AR1) was used to remove temporal autocorrelations and grand mean scaling was applied to normalize the time series to the percent change. Stimulus‐specific BOLD effects were estimated by convolving the stimulus trials with the canonical hemodynamic response function, with the onset timings set to mid‐point of the volume acquisition to give the smallest average timing error. Trials were modeled as epochs, corresponding to the duration of the respective sound file. Contrast images from each subject were combined into a group random effects analysis and compared in a series of *t*‐tests. Significant clusters were thresholded at FDR 0.05 corrected for multiple comparisons.

For RSA, GLMs were constructed using unsmoothed native space images that have been realigned and coregistered to the subject's MPRAGE. As RSA is based on comparing fine‐grained spatial patterns across conditions, the analyses were performed in native space to avoid the potential loss of information associated with normalizing the data to a template. To extract the patters of activity across the language‐processing regions for each participant, we used the inverse version of the native‐to‐stereotaxic transformation matrix to transform the bilateral frontotemporal language areas into each participant's native space (as before, the regions were anatomically defined using the WFU Pickatlas with the exception of FOP, which was defined based on published coordinates as noted above). This allowed us to determine the precise location of target regions in any given subject while keeping all the information contained in fine‐grained spatial activation patterns. Parameter estimates for each condition were then used to create the representational dissimilarity matrices (RDMs), which represent the correlation distance (1 – *r*, Pearson correlation across voxels) between activation patterns elicited by pairs of conditions. Each cell of an RDM represents the dissimilarity between activation patterns in two conditions. For each region, RDMs were averaged across participants and compared against theoretical models, also expressed as model RDMs. The match between the activation RDMs and model RDMs was tested by means of a second‐order correlation distance test, which assesses the correlation distance between these matrices [Kriegeskorte et al., 2008]. Statistical inference was assessed by a permutation test, with the key results also preserved after FDR correction for the number of regions tested in each model [Storey, 2002]. The correlation between two RDMs is assessed against a null‐hypothesis. The null hypothesis distribution of correlations was obtained by repeatedly randomizing the condition labels in one RDM and comparing it against the other. Finally, results are visualized using multidimensional scaling, where distances reflect similarities between activation RDMs and model RDMs.

## RESULTS

### Univariate Analyses

**Figure 1:**
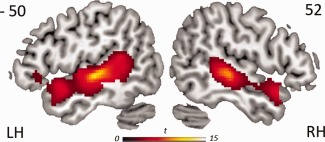
Significant activation for lexical processing (all words minus MuR), rendered onto the surface of a canonical brain. Clusters thresholded at *P* < 0.05 FDR corrected for multiple comparisons.

We first tested for the activity specifically related to speech‐driven lexical processing, by subtracting the MuR baseline from all speech stimuli. Consistent with the literature, lexical processing activated regions in bilateral superior and middle temporal lobes, and LIFG (Fig. 1 and Table S1, Supporting Information). Direct comparisons between the three different types of grammatical sequences (with the MuR acoustic baseline subtracted out) showed stronger activation for phrases over stems in bilateral middle and superior temporal areas (peaks at 56 0 −8; −58 −8 −6; and 52 −34 6, Table S2, Supporting Information), but no differences between stems and inflected forms or between inflected forms and phrases.

To test for possible effects of verb dominance we turned to a parametric analysis technique, where verb dominance score was entered as a parametric modulator for stems, inflected forms and phrases. There was evidence for modulation in left MTG for inflection‐related processing (peak activation in −60 −26 −4, cluster size 47 voxels), with more verb‐dominant inflected forms producing stronger activation. This effect, however, failed to reach corrected significance thresholds. Phrase‐related processing showed only a weak trend in the same direction (peak activation in −54 4 −2, cluster size 17 voxels). No modulation emerged in the right hemisphere, and there was no modulation for stem‐related processing in either hemisphere.

### Multivariate Analyses

**Figure 2:**
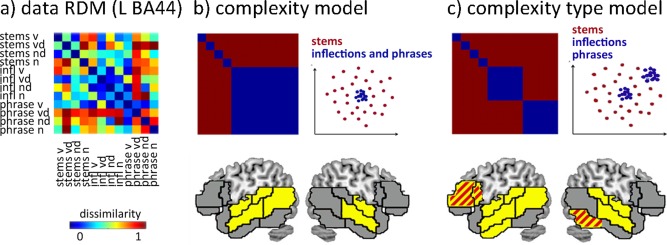
**a**) Activation RDM from L BA44: 12 × 12 matrix of correlation distances (one minus the correlation value) between activation patterns for each pair of conditions. RDMs are symmetrical across the diagonal. **b**) Upper left: model RDM coding for sensitivity to complexity processing, regardless of type. Blue indicates correlated activation patterns due to a shared property (presence of complexity), red indicates no correlation. Upper right: cartoon representation of the hypothesized distribution of the activations patterns in any given region. Each dot represents one activation pattern; the dissimilarity between them is shown as distance in 2D Euclidean space. Bottom: Brain regions that significantly correlate with this model (*P* < 0.05). **c**) A model RDM which codes for differential sensitivity to inflectional and phrasal complexity. Regions that significantly correlate with this model (*P* < 0.05) are shown in yellow. Red stripes indicate regions where this model fits significantly better than the general complexity model.

Multivariate RSA is sensitive to the informational patterning of neural activity [Kriegeskorte et al., 2008], and was used to perform more specific tests of the qualitative properties of grammatical computations in different areas. The analyses focused on the same set of bilateral language‐processing regions (BA44, BA45, BA47, FOP, superior, middle and inferior temporal gyri; temporal poles). For greater anatomical precision and consistent with evidence for different involvement of anterior and posterior temporal regions in grammatical processing [e.g., Friederici, 2011], superior, middle, and inferior temporal gyri were further split into anterior and posterior parts. Within each ROI, the activation pattern across all voxels for each condition was extracted, and correlated pairwise with the activation pattern for every other condition. The results are expressed as matrices of (dis)similarity between pairs of conditions (RDMs), with each cell of an RDM representing the correlation distance (1 – *r*) between activation patterns elicited by a pair of conditions. A sample activation RDM for L BA44 is given in Figure 2a. Each RDM was then compared against models (also expressed as RDMs) that represent specific hypotheses about the processing of grammatical complexity.

The first model (Fig. 2b) tested which regions show sensitivity to the processing of complex grammatical sequences, regardless of their type. This “general complexity” model assumes that any grammatically complex item creates an activation pattern that is similar to the pattern triggered by other complex items, but which is dissimilar to the pattern triggered by simple words. The results revealed a network of temporal regions, covering anterior STG and MTG bilaterally and posterior STG and MTG in the LH, and suggesting bilateral temporal involvement in the processing of grammatically complex sequences. Against this background of an implied common substrate for grammatically related processing, we then tested, using the “complexity type” model (Fig. 2c), for brain regions where the activation patterns for each type of complexity are distinct from each other (and where both are distinct from the stem items). The results showed an extended network, encompassing all the regions fitting the general complexity model, but adding L BA44, BA45, and anterior ITG, and R posterior ITG (for details see Table S3, Supporting Information). This implies that there is a large network of frontotemporal regions that are involved in grammatical processing, but which differentiate between the patterns of neural activity elicited by each type of complexity. Direct comparisons between the two models showed that the complexity type model fits significantly better than the general complexity model in L BA44, L BA45, and R pITG (indicated by red cross‐hatching in Fig. 2c).

**Figure 3:**
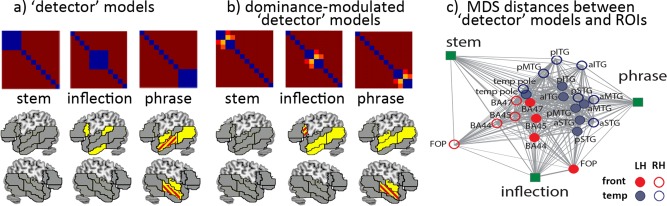
**a**) “Detector” models, coding for the processing of stems, inflected forms and phrases. Significant correlations for each model (*P* < 0.05) are shown in yellow. Red lines indicate significantly better fit of the phrase model over the inflection model. **b**) “Detector” models modulated by verb dominance. Red stripes denote regions of significantly better fit of inflections over phrases, and vice versa. **c**) Distances between regions and “detector” models in multidimensional space (MDS). Line area (length × thickness) specifies the distances, to compensate for distortions introduced by the projection from multidimensional space into 2D. Frontal regions are represented in red, temporal regions in blue; filled circles = LH, empty circles = RH

These results suggest a degree of functional differentiation within the language processing network, but do not indicate whether a specific type of complexity (inflectional or phrasal) predominates in any given region fitting the “complexity type” model. In the follow‐up analyses, we tested whether any specific brain regions could be predominantly associated with either inflectional or phrasal processing. To do this, we used a set of “detector” models, testing selectively for the processing of stems, inflected forms and phrases (Fig. 3). These are models that select for regions where a single type of process statistically dominates, relative to the other potential processing types. The first set of detector models (Fig. 3a) did not differentiate between conditions as a function of verb/noun dominance, giving equal weight to all four categories (verb unique, verb dominant, noun dominant, noun unique) within a type of grammatical sequence. They can, therefore, be viewed as asking a general question about different types of grammatical combination (inflectional or phrasal), and not about the internal structure of these processes (e.g., engaging verbal rather than nominal lexical representations).

The results (Fig. 3a) reveal a striking disjunction between the areas that best fit the inflectional and the phrasal models. No significant correlations were elicited by the stem model. Inflectional complexity correlates with a left‐lateralized set of regions: left BA44, pSTG, and aITG. Phrasal complexity, in contrast, correlates with a nonoverlapping bilateral network, encompassing anterior STG and MTG bilaterally, as well as L pMTG. Direct comparisons between inflection and phrase detector models showed a significantly better fit for the phrasal model in bilateral aMTG.

In a follow‐up analysis (Fig. 3b), we then asked whether verb/noun dominance modulates the observed effects, by weighting the detector models in each set according to the relative degrees of verb and noun dominance. These weights grouped together the verb unique and verb dominant sets (with values of 1.0 and 0.84, respectively) separately from the noun unique and the noun dominant sets (with values of 0.0 and 0.06). The primary effect of this is to make the detector models more sensitive to the noun/verb distinction, and to the potential representational and combinatorial consequences of this distinction. The results (Fig. 3b) revealed increased correlations for the dominance‐modulated inflection model relative to the simple detector model (Fig. 3a), but no changes to the pattern elicited by the phrasal model. In particular, apart from the preserved effects in L BA44 and L aITG, we now see inflectional model effects in L a/pMTG—also shared with the phrasal model. Consistent with the trend observed in the parametric analyses, this suggests that the grammatical category of the stem (verb/noun) modulates the combinatorial processes invoked by inflectional morphemes but not the computation of simple phrase structure.

Finally, plotting the distances in multidimensional space between detector models and activation patterns across all areas, Figure 3c confirms that L frontotemporal regions have the closest links to the inflectional model while bilateral temporal regions are closer to the phrasal model (for details see Table S4, Supporting Information).

## DISCUSSION

This experiment investigated the distribution and the nature of grammatical computations in the bilateral frontotemporal language network. While there is strong evidence that LH frontotemporal regions play a critical role in supporting grammatical computations, data from brain damaged patients suggest that some aspects of grammatical processing could be supported bilaterally. Here, we aimed to establish what are the processing capacities of the distributed bilateral network, and what specific types of grammatical computation drive the engagement of the left hemisphere circuit.

To this end, we compared the processing signatures for inflectionally complex words and minimal phrases, each requiring combinatorial linguistic analysis, against a background of grammatically simple words. The results revealed that grammatical processes engage a large bilateral frontotemporal network but with substantial differences in the way that inflectional and phrasal combination interfaces with this network. These differences arguably reflect distinguishable processing subsystems, which share L middle temporal representations relevant for lexically driven morphosyntactic processes, but have otherwise separable distributions.

The clearest separation of the neural substrates for inflectional and phrasal processing is provided by the generic “detector” RSA models (Fig. 3a), which look for the regions predominantly engaged either by the presence of an inflectional suffix (whether attached to a verb or noun stem) or by the presence of a simple noun or verb phrase. The inflectional model picks out LH regions (BA44 and pSTG) previously associated with inflectional complexity [Tyler et al., 2005], as well as the less frequently seen L anterior ITG. The phrasal model picks out a separate set of areas, engaging posterior and anterior MTG bilaterally. The second set of detector models, modulated by verb dominance and sensitive to the representational and combinatorial differences between verbs and nouns, modifies the distribution of LH areas that correlate with the inflectional model while not changing the distribution of bilateral areas correlating with the phrasal model. Instead of the discrete separation of the two models they now overlap in L anterior and posterior middle temporal regions, pointing to a shared basis for lexical interpretation.

What do these results reveal about the grammatical computational capacities of a distributed language processing network? Inflectional morphology is a core combinatorial grammatical device, where grammatical suffixes ({‐s},{‐ed},{‐ing} in English) attach to a stem to adjust it to the syntactic requirements of the environment (e.g., marking a verb for tense and aspect, or expressing the grammatical role of a nominal form). These inflectional cues influence the interpretation of both the stem and the sentential context to which the stem relates. The processing of inflected forms is, therefore, likely to trigger several interrelated computations, consistent with the findings that inflectional processing shares the underlying neurocognitive substrates of complex syntax, as well as its functional characteristics [Jackendoff, 1999]. At least some of these computations will reflect the fact that inflected words like *jumped* or *cats* are not stored lexical entries. Successful access to the lexical information conveyed by the stem and the grammatical information conveyed by the suffix (and their subsequent integration) requires morphophonological parsing and decomposition [Marslen‐Wilson and Tyler, 2007]. The dorsal circuit (BA44 and pSTG) picked out by the inflectional detector model is likely to be driven by these morphophonological parsing demands. Results consistent with this functional interpretation were reported by Tyler et al. [2005], who explicitly contrasted regularly and irregularly inflected verbs, which are equally linguistically complex but differ in the presence or absence of a phonologically separable inflectional suffix. The regularly inflected verbs (e.g., *played, walked*) evoked stronger activation than their irregular counterparts (e.g., *taught, broke*) in BA44 and in LSTG (and also RSTG), with similar patterns seen for any potentially inflected items (even nonwords, such as tade). This is also consistent with evidence from acquired aphasia, where nonfluent LH patients have difficulties with exactly this class of complex items [Tyler et al., 2002]. In a more recent study using MEG, Fonteneau et al. (in press) report time‐locked functional connectivity between BA44 and LpSTG that is specific to the presence of an inflectional morpheme.

The proposal for a dorsal circuit linking L STG to BA 44 (typically via the arcuate fasciculus) has long been part of models of the language system [e.g., Frederieci and Gierhan, 2013], and recent analyses by Rolheiser et al. [2011] suggest properties for this circuit that are consistent with the proposals here. Rolheiser et al. [2011] measured the performance of patients with LH damage on a range of tasks covering phonological, morphological, syntactic and semantic performance, and correlated these with relative degrees of damage to major dorsal and ventral white matter tracts. Damage to the arcuate fasciculus, implicating the LpSTG/BA44 circuit, most strongly affected tasks involving phonological or morphological segmentation, both in production and comprehension. This convergence of evidence from neuropsychology and from neuroimaging with unimpaired participants identifies a system that is closely adapted to the morphophonological parsing requirements associated with the presence of bound inflectional morphemes in the speech stream. The phrasal forms, in contrast, where the grammatical element (article or pronoun) is not phonologically integrated with the stem in the same way as an inflectional suffix, evidently do not make the same demands on the system supporting dynamic segmentation. Instead, we see a robust bilateral temporal pattern of correlation, primarily involving bilateral STG and MTG.

Modulating the inflectional model by verb dominance reveals how inflectional computations interact with the representational and combinatorial differences between verbs and nouns. Verbs have more complex lexical representations (argument structure) and carry more weight in grammatical structure building than nouns, as reflected in findings that they engage combinatorial processes more strongly than nouns when put in the appropriate grammatical environments [Longe et al., 2007; Tyler et al., 2008]. These differences emerged more clearly when we separated the activation patterns triggered by inflected verbs and inflected nouns using the dominance‐modulated model. Compared to the results seen in the “generic” inflectional model, the distribution of the engaged LH areas shifted to include L anterior and posterior MTG, in addition to the previously seen L BA44. Left MTG is commonly implicated in representing lexical knowledge and in mediating access to these representations [Hickok and Poeppel, 2007; Hagoort, 2013], and it is likely that the combinatorial interactions of verbal and nominal representations with L frontal areas differentially engage this region. The results suggest that the dominance‐modulated model indeed captures differences in the way grammatical combination interacts with lexical representations for verbs and nouns. Combined with the data from parametric analyses, this shows that inflected verbs engage combinatorial grammatical processes more strongly than inflected nouns in the left perisylvian network, consistent with earlier research.

Where our results diverge from standard accounts is in the bilateral temporal pattern seen for the processing of short phrases—most clearly delineated by the generic phrasal model—with no selective engagement of LIFG or L FOP, nor any evidence for inferior frontal activity bilaterally. In these strings, words from different grammatical categories (e.g., noun, verb, pronoun) combine following grammatical structure‐building constraints, and the comprehension of these structures requires consideration of this grammatical relationship, beyond simple activation of the lexical representations of the constituents. Critically, the short phrases used in this experiment are canonically ordered grammatical sequences, where the first element (a pronoun or an article) defines the grammatical properties of the subsequent element in a simple linear fashion. Computationally, they can be described in terms of deterministic left‐to‐right transitions between adjacent concatenated elements, which can be captured by regular FSG grammars [Fitch and Friederici, 2012]. As reviewed earlier, these types of computations and the processing of local phrase structures have been related to activity in the left ventral frontotemporal stream and the FOP in particular [Friederici et al., 2006a], but the available evidence provides only partial support for the strict left‐lateralization of these processes. Instead, studies often show bilateral temporal engagement for the processing of simple canonical sequences [Tyler et al., 2010], with the operculum activity reliably emerging in tasks that involve violations of these structures [Friederici et al., 2000a; Friederici et al., 2003; Friederici et al., 2006a]. This has raised questions about the link between operculum activity and grammatical computations, and suggests that it may reflect error detection and reanalysis instead [Caplan, 2007], consistent with the association found between the FOP activity and the decision‐making aspects of auditory perception [Binder et al., 2004]. The results here agree with this view: we saw no specific involvement of the FOP for the processing of simple canonical phrases under passive listening conditions. Instead, they seem to engage bilateral temporal structures only.

Neuropsychological data provide a background for the bihemispheric involvement in processing simple canonical sentences. Even if damage to left frontotemporal regions produces significant impairment in patients' ability to process complex syntactic structures [Caramazza and Zurif, 1976; Caplan et al., 1996], it does not necessarily affect their ability to understand simple canonical SVO sentences [Caramazza and Zurif, 1976; Tyler et al., 2011]. This points to the capacity of the intact RH structures (and potential residual LH functionality) to support simple, linear grammatical processes—sufficient for understanding canonical SVO sentences—but not the computations required for the processing of complex syntactic structures.

These results corroborate and extend the existing evidence for the critical role of a bihemispheric system in the lexical and pragmatic interpretation of spoken utterances [Bozic et al., 2010; Marslen‐Wilson and Tyler, 2007], by pointing to the capacity of this system to support the construction of local syntactic structures based on word category information, providing a basic scaffolding for interpretation. As the speech input unfolds, these simple structures enable the sequential integration of lexico‐semantic and prosodic information carried by the incoming linguistic elements.

The role of bilateral temporal structures in specifically syntactic processing (as distinct from lexico‐semantic and prosodic processes) has also been seen in earlier studies. Friederici et al. [2000], for example, compared the activations for spoken sentences and word‐lists, which are matched with respect to lexical information, and found stronger bilateral anterior temporal activation for sentences than word lists. Comparable results emerge from studies using written sentences [e.g., Vandenberghe et al., 2002], which remove the potential influences of prosodic cues to grammatical structure that are present in sentences but not word lists. To directly test the effects of prosody, Meyer et al. [2004] compared the activity triggered by correct but prosodically flattened sentences against sentences where the prosodic information was preserved but syntactic information was absent. The results showed stronger bilateral temporal activity for correct but prosodically flattened sentences, supporting the hypothesis about the syntactic nature of these activations.

Finally, we need to address the finding that these simple linear computations do not differ for verb and noun phrases, as revealed by the similarity of activation patterns seen for the “generic” and the dominance‐modulated phrasal models. If, as we suggest, the bilateral circuit indeed supports the linear groupings between adjacent elements, this is arguably what the predicted pattern of results would be. In the context of unfolding acoustic information, hearing a pronoun or an article is deterministic with respect to the grammatical properties of the subsequent element. This renders the combinatorial and representational differences between verbs and nouns less relevant, revealing instead a common underlying mechanism of simple constituent structure grouping.

These separable inflectional and phrasal subsystems thus appear to underlie different types of relational linking of successive elements as they are heard. In the case of the LH inflectional subsystem, these are the computations of short‐ and long‐distance grammatical relations and functions, and in the case of the bilateral phrasal subsystem they are the computations of constituent structure grouping. Jointly, these two mechanisms provide dynamic constraints on the interpretation of a spoken utterance as it unfolds and as its successive elements are mapped onto their lexical representations.

In conclusion, these data show a distribution of grammatical capacities between bilateral and left‐lateralized processing subsystems. The bilateral subsystem appears to support computations of local phrase structure and constituent structure grouping. This contrasts with the more complex grammatical computations associated with increased activity in the LH processing subsystem. A broader context for this distribution of computational capacities across the two subsystems comes from a developing neurobiological framework in which the key support for lexical, semantic, and pragmatic interpretation of auditory inputs comes from a distributed bihemispheric network, whose functional properties are traced back to the ancestral systems seen in nonhuman primates [Marslen‐Wilson et al., 2014]. Research suggests deep underlying parallels between bilateral networks for auditory object processing—including conspecific signals—in humans and macaques [e.g., Gil‐da‐Costa et al., 2006]. Additional evidence that non‐human primates can learn linear sequences of sounds defined by simple transitional probabilities between concatenated elements [Arnold and Zuberbühler, 2006; Fitch and Friederici, 2012; Wilson et al., 2013] suggest further parallels that may underpin bihemispheric linguistic capacities in the modern human.

A critical difference between the primate and the human brain is the set of LH structures and white matter connections that link posterior temporal to inferior frontal areas BA 44/45 [Rilling et al., 2008; Rolheiser et al., 2011]. In current models of language processing, these pathways are commonly associated with grammatical computations—most specifically with the processing of complex hierarchical structures and dependencies generated by supra‐regular grammars [Friederici, 2011] which non‐human primates are not capable of mastering [Fitch and Hauser, 2004]. This study provides support for the critical role of this LH frontotemporal network in complex grammatical processing, but in addition shows that grammatical computation may not be confined to the left hemisphere, and that the bihemispheric system plays a complementary role in supporting this key language function.

## Supporting information

Supplementary Information
