# Supplementary material for: Grammatical analysis as a distributed neurobiological function
Source: Hum Brain Mapp. 2014 Nov 24;36(3):1190–201. doi: 10.1002/hbm.22696 (PMC4365731; doi:10.1002/hbm.22696)
Supplement: Supplementary file 1 — Supplementary Information [file HBM-36-1190-s001.doc]

**Table S1**

*Regions that support lexical processing. Statistics of the clusters are shown thresholded at p<.05 FDR corrected for multiple comparisons. Throughout, the highest peaks within an extent are shown on subsequent lines, with the most significant shown in bold. The left temporal cluster extends into inferior frontal areas, with the most significant peak in this region marked with an asterisk.*

| *Regions* | *Extent Z* | | *Coordinates* | | | |
| --- | --- | --- | --- | --- | --- | --- |
| ***Words – MuR*** |  |  | *x* | *y* | *z* |  |
| **L MTG (BA21)** | **3694** | **6.76** | **-58** | **-28** | **0** |  |
| L STG (BA22) |  | 6.41 | -58 | -8 | 0 |  |
| L Superior Temporal Pole (BA38) |  | 5.49 | -56 | 8 | -10 |  |
| L Inferior Frontal Gyrus (BA 47)* |  | 2.87 | -46 | 28 | -2 |  |
| **R MTG (BA21)** | **2476** | **6.46** | **62** | **-24** | **-2** |  |
| R STG (BA22) |  | 5.17 | 62 | -6 | -8 |  |
| R Superior Temporal Pole (BA38) |  | 4.55 | 52 | 14 | -16 |  |

**Table S2**

***Direct comparisons between the lexical activation for phrases over stems (with the MuR acoustic baseline subtracted out). Statistics of the clusters are thresholded at p<.05 FDR corrected for multiple comparisons. a= anterior; p=posterior***

| *Regions* | *Extent Z* | | *Coordinates* | | | |
| --- | --- | --- | --- | --- | --- | --- |
| ***Phrases - Stems*** |  |  | *x* | *y* | *z* |  |
| **R aMTG (BA21)** | **411** | **4.62** | **56** | **0** | **-8** |  |
| **L aSTG (BA22)** | **1300** | **4.32** | **-58** | **-8** | **-6** |  |
| L pMTG (BA21)  L aMTG (BA21) |  | 4.09  3.67 | -60  -56 | -24  6 | 0  -16 |  |
| **R pSTG (BA22)**  R pMTG (BA21)  R pITG (BA20) | **533** | **4.05**  3.92  3.05 | **52**  52  50 | **-34**  -28  -22 | **6**  -4  -14 |  |
|  |  |  |  |  |  |  |

**Table S3**

*Table of correlation values (r) between the activity patterns in language-processing regions and the two models. Significant correlations are determined by means of a permutation test and denoted by bold (with significance level shown by asterisks, ** p<.01; * p<.05). Correlations that also reach FDR-corrected significance for multiple comparisons are marked in italic. A=anterior, p=posterior, FOP=frontal operculum*

| *region* | *complexity model* | *complexity types model* | |
| --- | --- | --- | --- |
| L BA44 | 0.10 | ***0.26**** |  |
| L BA45 | 0.10 | ***0.24**** |  |
| L BA47 | -0.06 | 0.09 |  |
| L FOP | 0.19 | 0.10 |  |
| L temp pole | -0.16 | 0.10 |  |
| L aSTG | **0.33*** | ***0.40***** |  |
| L pSTG | ***0.57***** | ***0.46***** |  |
| L aMTG | **0.45*** | ***0.51***** |  |
| L pMTG | ***0.40**** | ***0.42***** |  |
| L aITG | 0.15 | ***0.33**** |  |
| L pITG | 0.10 | *0.23* |  |
| R BA44 | -0.09 | 0.14 |  |
| R BA45 | -0.02 | 0.13 |  |
| R BA47 | 0.05 | 0.13 |  |
| R FOP | -0.04 | -0.04 |  |
| R temp pole | -0.11 | 0.10 |  |
| R aSTG | ***0.53***** | ***0.51***** |  |
| R pSTG | 0.30 | ***0.31**** |  |
| R aMTG | **0.38*** | ***0.37***** |  |
| R pMTG | 0.15 | 0.16 |  |
| R aITG | 0.03 | 0.15 |  |
| R pITG | 0.01 | ***0.26**** |  |

**Table S4**

*Table of correlation values (r) between the regions and the ‘detector’ models and their dominance-modulated variants. Significant correlations are determined by means of a permutation test and denoted by bold (with significance level shown by asterisks, ** p<.01; * p<.05). Correlations that also reach FDR-corrected significance for multiple comparisons are marked in italic.*

| *region* | *stem* | *stem dominance* | *inflection* | *inflection dominance* | *phrase* | *phrase dominance* |
| --- | --- | --- | --- | --- | --- | --- |
| L BA44 | -0.03 | 0.02 | **0.32*** | ***0.34***** | 0.04 | -0.01 |
| L BA45 | 0.00 | 0.06 | 0.16 | 0.14 | 0.16 | 0.07 |
| L BA47 | 0.16 | 0.18 | 0.20 | 0.21 | -0.08 | -0.19 |
| L FOP | -0.29 | -0.22 | 0.15 | 0.20 | -0.01 | -0.04 |
| L temp pole | 0.22 | 0.21 | 0.08 | 0.07 | 0.05 | -0.04 |
| L aSTG | -0.05 | -0.06 | 0.20 | 0.20 | ***0.34**** | **0.27*** |
| L pSTG | -0.23 | -0.15 | **0.32*** | 0.26 | 0.30 | 0.22 |
| L aMTG | -0.24 | -0.18 | 0.22 | **0.27*** | ***0.46***** | ***0.40***** |
| L pMTG | -0.14 | -0.07 | 0.25 | **0.27*** | ***0.32**** | **0.26*** |
| L aITG | -0.06 | -0.02 | **0.27*** | **0.28*** | 0.17 | 0.11 |
| L pITG | 0.02 | 0.04 | 0.15 | 0.21 | 0.15 | 0.08 |
| R BA44 | 0.16 | 0.23 | 0.17 | 0.10 | 0.01 | -0.06 |
| R BA45 | 0.07 | 0.16 | 0.07 | 0.01 | 0.10 | 0.02 |
| R BA47 | 0.06 | 0.16 | 0.17 | 0.08 | 0.01 | -0.07 |
| R FOP | 0.21 | **0.29*** | 0.09 | 0.09 | -0.14 | -0.16 |
| R temp pole | 0.15 | 0.23 | 0.02 | 0.06 | 0.11 | 0.04 |
| R aSTG | -0.24 | -0.17 | 0.26 | 0.23 | ***0.42***** | ***0.36***** |
| R pSTG | -0.01 | 0.07 | 0.17 | 0.14 | 0.24 | 0.16 |
| R aMTG | -0.04 | 0.03 | 0.05 | 0.03 | ***0.44***** | ***0.38***** |
| R pMTG | 0.04 | 0.10 | 0.09 | 0.12 | 0.13 | 0.05 |
| R aITG | 0.03 | 0.12 | 0.09 | 0.03 | 0.11 | 0.03 |
| R pITG | 0.12 | 0.20 | 0.12 | 0.08 | 0.24 | 0.15 |
